# Supplementary material for: Immunomodulatory Effects of Atractylodes lancea in Healthy Volunteers with Dosage Prediction for Cholangiocarcinoma Therapy: A Modelling Approach
Source: Pharmaceuticals (Basel). 2025 Jan 31;18(2):198. doi: 10.3390/ph18020198 (PMC11860138; doi:10.3390/ph18020198)
Supplement: Supplementary file 1 [file pharmaceuticals-18-00198-s001.zip › pharmaceuticals-3415679-supplementary.pdf]

Supplementary

Figure S1 A comparison of atractylodin plasma concentration-time profiles following a single dose of 1,000 mg of *Atractylodes Lancea* (Thunb) DC on day 1 between predicted values and clinical data [5].

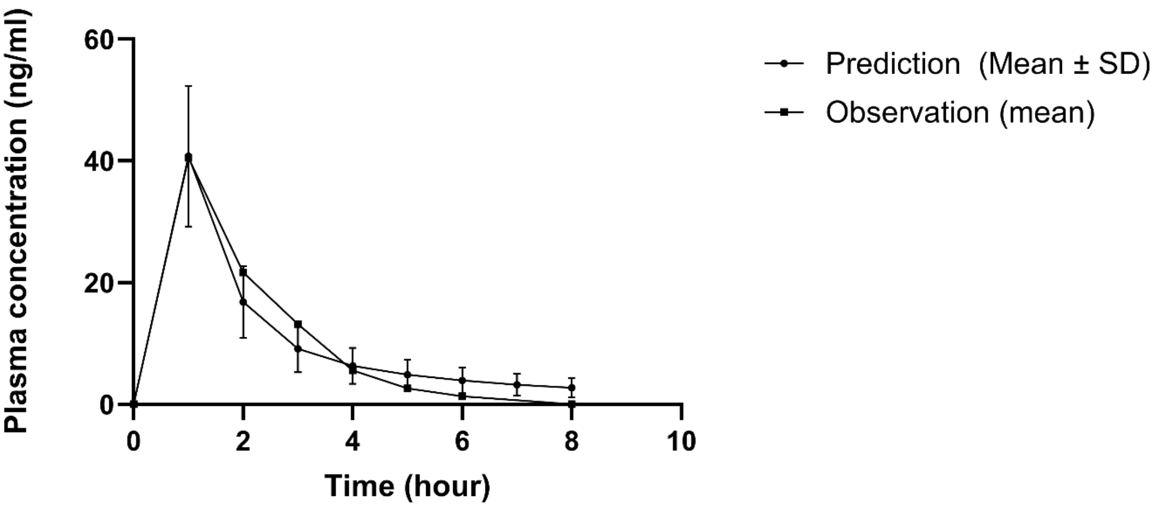

Figure S2 A comparison of atractylodin plasma concentration-time profiles following multiple doses of 1,000 mg of *Atractylodes Lancea* (Thunb) DC on day 1 between predicted values and clinical data [5].

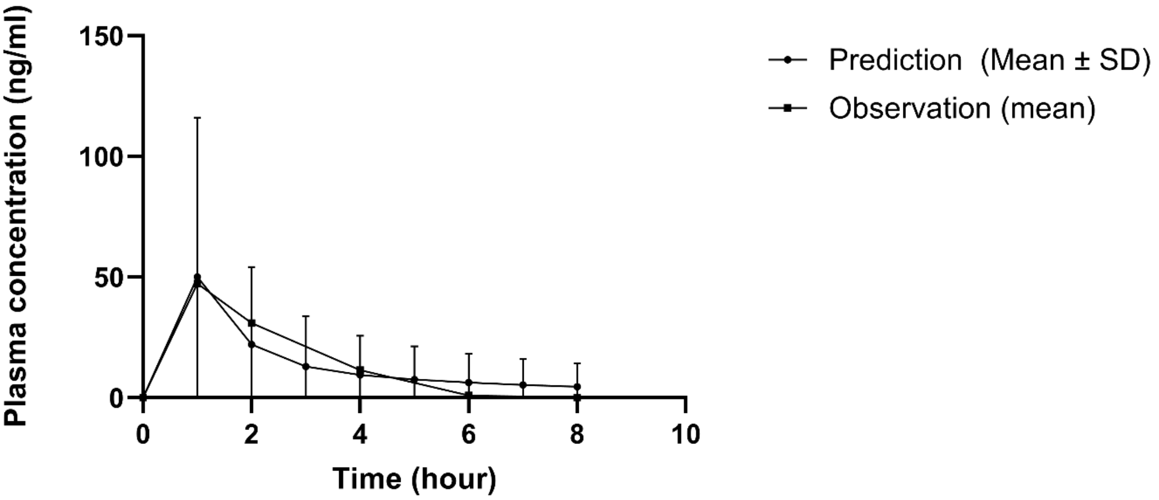

Figure S3 A comparison of atractylodin plasma concentration-time profiles following multiple doses of 1,000 mg of *Atractylodes Lancea* (Thunb) DC on day 21 between predicted values and clinical data [5].

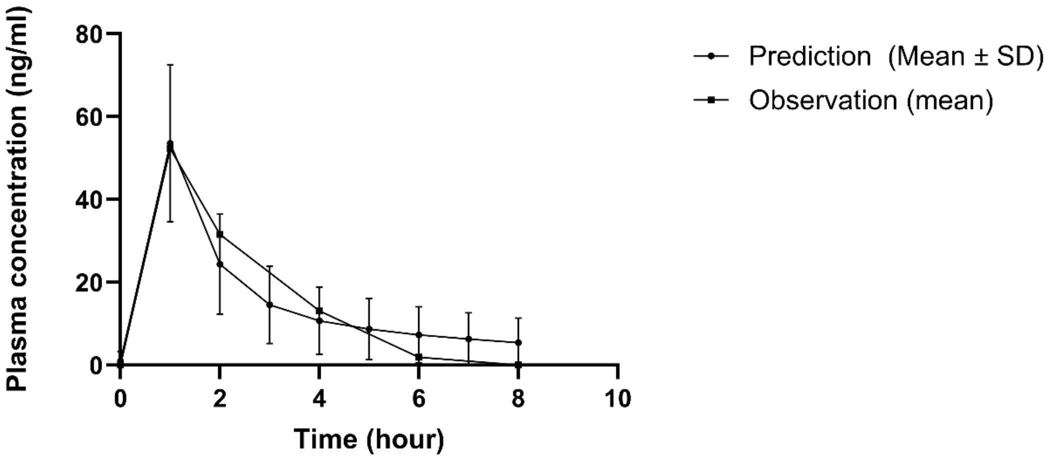

Table S1. Model parameters

**Number**	**Parameter**	**Value**	**Reference**
**1**	Fraction of unbound drug (f_u)	0.039	In house
**2**	pKa	9.63	In house
**3**	Log D	4.91	Pub-chem
**4**	Blood-to-plasma partition ratio (R_{bp})	1.03	In house
**5**	PSA	13.1	Pub-Chem
**6**	HBD	1	Pub-Chem
**7**	Absolute bioavailability (F_{bio})	0.8	1
**8**	Clearance *in vivo* (L/h/kg)	6.26	1
**9**	Solubility (µg/ml) (PBS pH 6.8, SIF)	0.11±0.003	In house

Table S2. A statistical analysis of circulating cytokines following successive doses of 1,000 mg of AL administered for 21 days

**Treated group**				
**Cytokines**	**0 hr. versus 24 hr.**	**0 hr. versus 48 hr.**	**0 hr. versus day 7**	**0 hr. versus day 14**
IL-2	P=0.2121*	P=0.306*	P<0.0001*	P<0.001*
IL-4	P=0.2524*	P=0.7948*	P=0.0002*	P=0.0007*
IL-6	P=0.0637*	P=0.0826*	P=0.0353*	P=0.1303*
IL-10	P=0.9853 (t=0.018, df=19)	P=0.87 (t=0.164, df=19)	P=0.0032 (t=3.378, df=19)	P=0.0566 (t=2.03, df=19)
IL-17A	P=0.0002*	P=0.0188*	P=0.0008	P=0.0148
TNF-α	P=0.063 (t=1.972, df=19)	P=0.529 (t=0.64, df=19)	P<0.0001 (t=4.94, df=19)	P=0.164 (t=1.448, df=19)
IFN-γ	P=0.044 (t=2.154, df=19)	P=0.0362*	P=0.1211*	P=0.0148 (t=2.68, df=19)
**Untreated (Placebo) group**				
IL-2	P=0.875*	P=0.625*	P=0.125*	P=0.125*
IL-4	P=0.524 (t=0.72, df=3)	P=0.834 (t=0.23, df=3)	P=0.07 (t=2.76, df=3)	P=0.07 (t=2.76, df=3)

IL-6	P=0.145 (t=1.95, df=3)	P=0.35 (t=1.13, df=3)	P=0.11 (t=2.22, df=3)	P=0.543 (t=0.69, df=3)
IL-10	P=0.375*	P=0.25*	P=0.125*	P=0.625*
IL-17A	P=0.875*	P=0.5*	P=0.875*	P=0.125*
TNF-α	P=0.81 (t=0.26, df=3)	P=0.23 (t=1.51, df=3)	P=0.123 (t=2.59, df=2)	P=0.24 (t=1.47, df=3)
IFN-γ	P=0.88 (t=0.156, df=3)	P=0.28 (t=1.32, df=3)	P<0.0001 (t=96.85, df=3)	P=0.28 (t=1.32, df=3)

*Wilcoxon matched-pairs signed rank test; AL: Atractylodes Lancea (Thunb) D.C.; IL: interleukin; IFN-γ: interferon-gamma; TNF-α: tumor-necrosis factor alpha

Table S3. A statistical analysis of peripheral blood immune cells, SII index, NLR, LMR, PLR, and SII index following daily doses of 1,000 mg of AL administered for 21 days.

**Treated group**								
**Peripheral blood immune cells**	**0hr versus 24hr**	**0hr versus 48hr**	**0hr versus day 4**	**0hr versus day 7**	**0hr versus day 14**	**0hr versus day 21**	**0hr versus day 22**	**0hr versus day 23**
Lymphocytes	P=0.045 (t=2.15, df=18)	P=0.056 (t=2.04, df=18)	NA	P=0.023*	P=0.004 (t=3.29, df=18)	P=0.01 (t=2.87, df=18)	P=0.0008 (t=4.02, df=18)	P=0.06 (t=2.01, df=18)
T cells	P<0.0001 (t=5.74, df=18)	P=0.024 (t=2.47, df=18)	NA	P<0.0001 (t=6.16, df=18)	P<0.0001 (t=7.37, df=18)	P<0.0001 (t=5.34, df=18)	P=0.0013 (t=3.81, df=18)	P<0.0001 (t=6.39, df=18)
B cells	P<0.0001 (t=6.29, df=18)	P=0.056 (t=2.04, df=18)	NA	P<0.0001 (t=8.51, df=18)	P<0.0001 (t=7.57, df=18)	P=0.168 (t=1.44, df=18)	P=0.008 (t=2.97, df=18)	P=0.001 (t=3.88, df=18)
NK cells	P<0.0001 (t=13.49, df=18)	P<0.0001 (t=5.17, df=18)	NA	P<0.0001 (t=15, df=18)	P<0.0001 (t=13.18, df=18)	P=0.0005 (t=4.28, df=18)	P<0.0001 (t=10.51, df=18)	P<0.0001 (t=9.03, df=18)
CD4+	P<0.0001 (t=10.84, df=18)	P<0.0001 (t=9.47, df=18)	NA	P=0.0002 (t=4.75, df=18)	P=0.13 (t=1.58, df=18)	P<0.0001 (t=6.23, df=18)	P<0.0001 (t=5.27, df=18)	P=0.0001 (t=4.82, df=18)
CD8+	P=0.0012 (t=3.83, df=18)	P=0.112 (t=1.67, df=18)	NA	P=0.08 (t=1.83, df=18)	P=0.0006 (t=4.13, df=18)	P<0.0001 (t=5.05, df=18)	P=0.85 (t=0.18, df=18)	P=0.028 (t=2.40, df=18)
CD4+/CD8+ ratios	P=0.036*	P=0.59*	NA	P=0.19*	P=0.26*	P=0.08*	P=0.0008*	P=0.08*
NLR	NA	NA	P=0.26 (t=1.16, df=19)	NA	NA	NA	P=0.17 (t=1.42, df=18)	NA
LMR	NA	NA	P=0.014 (t=2.7	NA	NA	NA	P=0.11 (t=1.69, df=18)	NA

			0,<br>df=19<br>)					
PLR	NA	NA	P=0.3<br>9*	NA	NA	NA	P=0.59*	NA
SII index	NA	NA	P=0.3<br>9*	NA	NA	NA	P>0.999*	NA
**Untreated (Placebo) group**								
Lymphocytes	P=0.97<br>(t=0.04,<br>df=3)	P=0.98<br>(t=0.03,<br>df=3)	NA	P=0.68<br>(t=0.45,<br>df=3)	P=0.61<br>(t=0.57,<br>df=3)	P=0.61<br>(t=0.57,<br>df=3)	P=0.70<br>(t=0.41,<br>df=3)	P=0.12<br>(t=2.15,<br>df=3)
T cells	P=0.41<br>(t=1.03,<br>df=2)	P=0.74<br>(t=0.37,<br>df=2)	NA	NA	P=0.23<br>(t=1.68,<br>df=2)	P=0.028<br>(t=5.78,<br>df=2)	P=0.004<br>(t=14.90,<br>df=2)	P=0.007<br>(t=11.55,<br>df=2)
B cells	P=0.036<br>(t=3.61,<br>df=3)	P=0.22<br>(t=1.54,<br>df=3)	NA	P=0.66<br>(t=0.48,<br>df=3)	P=0.83<br>(t=0.23,<br>df=3)	P=0.39<br>(t=0.99,<br>df=3)	P=0.085<br>(t=2.53,<br>df=3)	P=0.047<br>(t=3.25,<br>df=3)
NK cells	P=0.04<br>(t=3.5,<br>df=3)	P=0.04<br>(t=3.5,<br>df=3)	NA	P=0.002<br>(t=10.04,<br>df=3)	P=0.018<br>(t=4.68,<br>df=3)	P=0.0027<br>(t=9.17,<br>df=3)	P=0.0003<br>(t=20.14,<br>df=3)	P=0.0007<br>(t=14.89,<br>df=3)
CD4+	P=0.087<br>(t=2.51,<br>df=3)	P=0.26<br>(t=1.34,<br>df=3)	NA	P=0.004<br>(t=7.8,<br>df=3)	P=0.02<br>(t=4.37,<br>df=3)	P=0.0018<br>(t=10.52,<br>df=3)	P=0.001<br>(t=12.31,<br>df=3)	P=0.0003<br>(t=18.91,<br>df=3)
CD8+	P=0.35<br>(t=1.10,<br>df=3)	P=0.45<br>(t=0.86,<br>df=3)	NA	P=0.56<br>(t=0.65,<br>df=3)	P=0.65<br>(t=0.49,<br>df=3)	P=0.044<br>(t=3.36,<br>df=3)	P=0.33<br>(t=1.14,<br>df=3)	P=0.004<br>(t=8.02,<br>df=3)
CD4+/CD8+<br>ratio	P=0.40<br>(t=0.97,<br>df=3)	P=0.52<br>(t=0.73,<br>df=3)	NA	P=0.37<br>(t=1.06,<br>df=3)	P=0.44<br>(t=0.89,<br>df=3)	P=0.27<br>(t=1.34,<br>df=3)	P=0.33<br>(t=1.17,<br>df=3)	P=0.41<br>(t=0.96,<br>df=3)
NLR	NA	NA	P=0.8<br>3<br>(t=0.2<br>4,<br>df=3)	NA	NA	NA	P=0.25*	NA
LMR	NA	NA	P=0.8<br>0<br>(t=0.2<br>7,<br>df=3)	NA	NA	NA	P=0.047<br>(t=3.24,<br>df=3)	NA
PLR	NA	NA	P=0.7<br>7<br>(t=0.3<br>1,<br>df=3)	NA	NA	NA	P=0.42<br>(t=0.94,<br>df=3)	NA
SII index	NA	NA	P=0.7<br>87<br>(t=0.3<br>6,<br>df=3)	NA	NA	NA	P=0.27<br>(t=1.35,<br>df=3)	NA

*Wilcoxon matched-pairs signed rank test; LMR: Lymphocyte-to-monocyte Ratio; NA: Not applicable; NK: Natural Killer; NLR: Neutrophil-to-lymphocyte Ratio; PLR: Platelet-to-lymphocyte Ratio; SII index: systemic inflammatory-immune response index.

Table S4. Model validation

Number	Parameter	AAFEs
All Group		
1	AUC	1.10
2	C_{max}	1.06
3	V_z/F	1.34
4	CL/F	1.13
5	$T_{1/2}$ (h)	1.22
AAFEs (Group 1; Day 1)	1.04	
Group 1 (Day 1)		
6	AUC	1.02
7	C_{max}	1.14
8	V_z/F	1.02
9	CL/F	1.03
10	$T_{1/2}$	1.00
Group 2 (Day 1)		
11	AUC	1.19
12	C_{max}	1.03
13	V_z/F	1.49
14	CL/F	1.28
15	$T_{1/2}$	1.27
AAFEs (Group 2; Day 1)	1.24	
Group 2 (Day 21)		
16	AUC	1.11
17	C_{max}	1.00
18	V_z/F	1.60
19	CL/F	1.22
20	$T_{1/2}$	1.42
AAFEs (Group 2; Day 21)	1.25	
AAFEs (All)	1.17	

AUC: area under the plasma concentration-time curve; C_{max}: maximum plasma concentration; Vz/F: volume of distribution; CL/F: apparent clearance; T1/2: terminal half-life.
